# A High Vitamin C Micronutrient Supplement Is Unable to Attenuate Inflammation in People with Metabolic Syndrome but May Improve Metabolic Health Indices: A Randomised Controlled Trial

**DOI:** 10.3390/antiox13040404

**Published:** 2024-03-28

**Authors:** Emma Vlasiuk, Masuma Zawari, Rebekah Whitehead, Jonathan Williman, Anitra C. Carr

**Affiliations:** 1 Nutrition in Medicine Research Group, Department of Pathology and Biomedical Science, University of Otago, Christchurch 8011, New Zealand; emma.vlasiuk@otago.ac.nz (E.V.); masuma.zawari@otago.ac.nz (M.Z.); whibe325@student.otago.ac.nz (R.W.); 2 Department of Population Health, University of Otago, Christchurch 8011, New Zealand; jonathan.williman@otago.ac.nz

**Keywords:** metabolic syndrome, obesity, inflammation, C-reactive protein, vitamin C, micronutrients, glycaemic indices, insulin sensitivity, metabolic severity score

## Abstract

Chronic low-grade inflammation is a characteristic of people with metabolic syndrome and is thought to contribute to the condition progressing to the more severe type 2 diabetes and cardiovascular disease (CVD). The aim was to carry out a double-blind randomised placebo-controlled trial in people with metabolic syndrome to determine if supplementation with a micronutrient formula containing 1000 mg/d vitamin C could attenuate inflammation in people with metabolic syndrome. We recruited 72 adults aged a median of 52 years with metabolic syndrome, defined as obesity (based on waist circumference or BMI), and at least two of hyperglycaemia, raised triglycerides, lowered HDL cholesterol, hypertension, or taking medications for these conditions. A further inclusion criteria comprised C-reactive protein (CRP) concentrations ≥ 3 mg/L, i.e., high risk of CVD. The participants were randomised to daily micronutrient formula (*n* = 37) or matched placebo control (*n* = 35) for 12 weeks. The primary outcome was change in CRP concentrations and secondary outcomes included changes in vitamin C concentrations, pro-inflammatory cytokines (IL-6, TNFα), oxidative stress marker (F_2_isoprostanes), glycaemic indices (glucose, insulin, HbA1c), lipid markers (triglycerides, LDL and HDL cholesterol), anthropometric parameters (weight, BMI), insulin resistance and insulin sensitivity, and metabolic severity score. The participants had a low median (Q1, Q3) vitamin C status of 29 (15, 41) µmol/L and a high proportion of hypovitaminosis C (38%) and outright deficiency (19%). Following 12 weeks of micronutrient supplementation, at least 70% of the participants reached adequate vitamin C status (≥50 µmol/L), however, there was no change in CRP concentrations relative to the placebo group (Δ−0.3 [95%CI −2.7, 2.1] mg/L, *p* = 0.8). Similar trends were observed for IL-6, TNFα and F_2_isoprostanes (*p* > 0.05). Instead, there were small improvements in BMI, fasting glucose and HbA1c concentrations, insulin sensitivity and metabolic severity score in the micronutrient group relative to placebo (*p* < 0.05). Overall, 12-week micronutrient supplementation was unable to mitigate systemic inflammation in people with metabolic syndrome but may improve several metabolic health indices.

## 1. Introduction

Metabolic syndrome is a chronic condition comprising a constellation of cardiometabolic risk factors characterised by abdominal obesity, insulin resistance, hypertension and dyslipidaemia [1]. Metabolic syndrome has become a major public health challenge worldwide, estimated to affect 20–25 per cent of the world’s adult population, increasing to 30–40% by age 65. It is a risk factor for more severe chronic diseases such as type 2 diabetes mellitus and various cardiovascular diseases (CVD); those with metabolic syndrome have a five-fold greater risk of developing type 2 diabetes and are three times as likely to have a heart attack or stroke compared with people without the syndrome [2]. Obesity is a major driving force behind metabolic syndrome. The World Health Organisation estimated that in 2016, 39% of the world’s population was overweight, and 13% was obese [3], with the risk of developing metabolic syndrome increasing strikingly above a BMI of 25. In a recent study, Park et al. [4] reported a prevalence of metabolic syndrome in 5%, 23%, and 60% of normal-weight, overweight, and obese men in the US population. As such, the likelihood of a further increase in metabolic syndrome can be anticipated because of projections of a greater prevalence of obesity in the future [5]. As such, there is urgency for the implementation of better approaches towards preventing progression of the syndrome to the more severe conditions of type 2 diabetes and CVD.

Inflammation is believed to be a driver of metabolic syndrome [6], with the condition displaying enhanced secretion of pro-inflammatory cytokines (e.g., interleukin-6; IL-6, and tumour necrosis factor α; TNFα) from adipose tissue and leukocytes [7]. Activation of pro-inflammatory signalling pathways leads to a condition of chronic low-grade inflammation and contributes to insulin resistance and vascular dysfunction [8,9]. Pro-inflammatory cytokines (e.g., IL-6) also stimulate the release of acute-phase reactants, such as C-reactive protein (CRP), which is a commonly measured marker of systemic inflammation and a known risk factor for CVD. Oxidative stress, an imbalance between the generation of reactive oxygen species and the ability of endogenous antioxidants to combat this, is closely associated with inflammatory processes and is implicated in the aetiology of metabolic syndrome [10]. In line with the emerging evidence that inflammation and oxidative stress play a significant role in the pathophysiology of metabolic syndrome, there is a growing interest in employing anti-inflammatory and antioxidant agents against metabolic dysregulation.

Vitamin C (ascorbate) is a potent antioxidant with anti-inflammatory properties [11]. Because humans have lost the ability to synthesise vitamin C endogenously, we must obtain it through our diet, particularly from fresh fruit and vegetables. Epidemiological research has highlighted that people with metabolic syndrome have lower vitamin C status than healthy controls [12], and higher baseline vitamin C status has been associated with a lower risk of metabolic syndrome [13,14]. Obesity is also a risk factor for low vitamin C status, with obese people requiring higher intakes to reach equivalent status to those of average weight [15,16]. Research has previously shown decreased vitamin C status in people with prediabetes or metabolic syndrome and this was inversely associated with obesity [17,18,19]. The lower vitamin C status was despite comparable dietary intakes of the vitamin [17,18,19], suggesting a higher utilisation of the vitamin in people with metabolic syndrome, possibly due to the inflammatory and oxidative stress processes occurring during the disease process. As such, targeting these processes with anti-inflammatory and antioxidant nutrients may help attenuate progression of metabolic syndrome.

Several studies have assessed the effects of vitamin C/antioxidant supplementation on inflammatory biomarkers in obesity and/or diabetes [20,21,22]. One showed decreased CRP and IL-6 concentrations relative to control, while the other studies showed no effect of intervention [21,22]. As far as we are aware, the effects of vitamin C supplementation on markers of inflammation in people with metabolic syndrome have not yet been assessed. People who are vitamin-C-deficient are often also deficient in other important micronutrients which have been shown to have anti-inflammatory and antioxidant effects, particularly in obesity [23,24,25,26]. The aim of our study was to assess the effects of a high vitamin C micronutrient supplement on inflammation in people with metabolic syndrome. The objective was to enroll participants with metabolic syndrome plus elevated CRP concentrations of ≥3 mg/L, indicative of a high risk for CVD, into a double-blind randomized placebo-controlled trial of a high vitamin C micronutrient supplement versus placebo control for 12 weeks duration. We hypothesized that administration of vitamin C (1000 mg/d) with other selected micronutrients would decrease systemic inflammation (i.e., CRP concentrations) in people with metabolic syndrome, thus providing a window of opportunity to potentially reverse or halt progression of the condition to the more severe cardiometabolic diseases.

## 2. Materials and Methods

### 2.1. Study Design

This was a double-blinded randomised placebo-controlled trial designed to assess the impact of daily supplementation with a high vitamin C micronutrient supplement for 12 weeks on inflammation in people with metabolic syndrome. The trial received ethical approval from the New Zealand Southern Health and Disability Ethics Committee (#21/STH/43, 8 April 2021) and was registered with the Australian New Zealand Clinical Trials Registry (https://www.anzctr.org.au, #ACTRN12621000678897, 3 June 2021). All participants provided written informed consent to participate in the study which was conducted according to the principles of the Declaration of Helsinki at the University of Otago, Christchurch, New Zealand, with rolling recruitment occurring between June 2021 and June 2023.

The primary study outcome was the effect of intervention on C-reactive protein (CRP) concentrations after 12 weeks of supplementation. Secondary outcomes included the effect of intervention on nutrient markers (vitamin C), pro-inflammatory cytokines (TNFα, IL-6), oxidative stress markers (F_2_isoprostanes), complete cell counts, glycaemic markers (glucose, insulin, HbA1c), lipid markers (triglycerides, total cholesterol, HDL cholesterol, LDL cholesterol), anthropometric parameters (weight, BMI, waist circumference, waist/hip ratio, blood pressure), homeostatic model assessment for insulin resistance (HOMA-IR), insulin sensitivity (HOMA%S), ß-cell activity (HOMA%B), and metabolic severity z score. The participants’ physical activities and dietary intakes were also monitored.

### 2.2. Study Participants

Participants were recruited through the local community, primarily via social media advertising. Following initial contact with the study coordinator, the participants attended a screening clinic to assess eligibility for the study. The International Diabetes Federation consensus definition of metabolic syndrome was used [27]. The inclusion criteria comprised adult males and females aged ≥18 years, central/abdominal obesity (i.e., waist circumference ≥94 cm for males, ≥80 cm for females or BMI ≥30 kg/m^2^) and either (a) hyperglycemia (i.e., fasting glucose ≥5.6 mmol/L or taking medication for this condition) or (b) raised triglycerides (i.e., TG ≥1.7 mmol/L or taking medication for this condition) or (c) lowered HDL (HDL <1.0 mmol/L males, <1.3 mmol/L females or taking medication for this condition) or (d) hypertension (i.e., blood pressure >130 systolic or >86 diastolic or taking medication for this condition). A further inclusion criteria comprised CRP concentrations ≥3 mg/L (i.e., high risk of CVD). Exclusion criteria comprised acute illness within the previous two weeks, taking anti-inflammatory medication (e.g., corticosteroids), suffering from inflammatory conditions (e.g., active malignancy, rheumatoid arthritis, lupus, inflammatory bowel disease), pregnant or breastfeeding or taking micronutrient (vitamin/mineral) supplements (unless willing to cease taking these for at least two weeks prior to beginning the study). Participants were asked to maintain their usual diets and physical activity and not to alter their lifestyle during the interventional period.

Power calculations indicated that a sample size of 70 (35 per group) would provide 80% power at a two-sided alpha of 0.05 to detect an absolute difference between the groups of 1.5 mg/L CRP (mean control = 4.5, i.e., high risk of CVD; mean treatment = 3.0, i.e., average risk of CVD), assuming an SD of 2.2 mg/L, based on a US study tracking CRP in 8900 individuals with initially elevated concentrations [28]. This comprises a large effect size of 0.68. Additional participants were recruited upon withdrawal of participants during the study to ensure that 70 people completed the 12-week study period.

### 2.3. Randomisation and Intervention

Allocation was concealed by using a pre-specified computer-generated random block size randomization list prepared by the study statistician. Participants were randomised (1:1) to receive either one micronutrient tablet or one placebo tablet daily. Treatment and placebo tablets were in identical containers and allocated using the spreadsheet provided by the biostatistician in the order participants entered the trial. Treatment/placebo were dispensed by the study coordinator who was blinded as to which arm the participants were allocated.

The intervention comprised an effervescent tablet containing 1000 mg vitamin C and other essential vitamins and minerals (at doses 0.6 to 6 times the NZ dietary recommended intake [29]; see Table 1 for composition). The inactive placebo tablet was closely matched for appearance and flavour (Bayer Consumer Care, Basel, Switzerland). Participants were provided with sufficient tablets for 12 weeks intervention. Compliance with the intervention was determined by counting returned tablets and confirmed by measuring plasma vitamin C concentrations.

### 2.4. Data and Sample Collection

At the screening visit, the following data were collected: participant demographics (age, gender, ethnicity), anthropometric parameters, relevant past medical history, comorbidities and medications. A fasting blood sample was also collected to determine CRP, glucose, triglycerides and HDL concentrations. The eligible participants attended study clinics at baseline (week 0), and weeks 6 and 12. At the clinics, the study coordinator assessed anthropometric measures (height using a stadiometer, waist and hip circumference using a tape measure, and weight using a TANITA SC-300 body composition analyser), blood pressure using a sphygmomanometer, physical activity, and dietary intake over the previous 24 h. Fasting blood (lithium heparin and EDTA tubes) and spot urine samples were collected for analysis of the inflammatory, oxidative, nutritive, glycaemic and lipid markers (as described below). Study data were managed via a REDCap^®^ database.

### 2.5. Laboratory Assessments

The fasting blood and urine samples were placed on ice immediately, and processed within one hour for analysis of routine biomarkers. The remainder of the plasma and isolated leukocytes were stored at −80 °C for analysis of study-specific biomarkers as described below. Routine inflammatory and infection markers (high sensitivity CRP, blood cell counts), glycaemic markers (glucose, insulin, HbA1c), lipid markers (triglycerides, total cholesterol, HDL-C, LDL-C) and urine creatinine were assessed by Canterbury Health Laboratories, an International Accreditation New Zealand (IANZ) laboratory. Plasma vitamin C assessments were carried out using the gold standard method of HPLC [30]. Samples were stabilized with acid and a metal chelator prior to storage at −80 °C to attenuate ex vivo oxidation [31]. Plasma TNFα and IL-6 concentrations were determined using commercial ELISA kits (ELISA MAX^TM^ Deluxe Sets, BioLegend, San Diego, CA, USA). Urinary F_2_-isoprostanes concentrations were determined using a commercial ELISA kit (STAT-8-isoprostane ELISA Kit, Cayman Chemical, Ann Arbor, MI, USA). These were standardised to urinary creatinine concentrations. Butylated hydroxytoluene (BHT) was added to urine samples prior to storage at −80 °C to attenuate ex vivo oxidation. Protein carbonyl ELISAs were trialled in a subgroup of participants at baseline (*n* = 11), as another potential marker of oxidative stress [32]; however, these did not differ significantly from healthy controls (mean of 0.243 vs. 0.259 nmol/mg protein, respectively), and so were not pursued further.

### 2.6. HOMA Indices and MetS Severity z Score

Homeostatic Model Assessment of insulin resistance (HOMA-IR) scores measure the presence and extent of insulin resistance and were calculated using the University of Oxford Diabetes Trials Unit HOMA2 calculator (version 2.2.4) [33]. Insulin sensitivity (HOMA%S) and ß-cell activity (HOMA%B) were also calculated. Scores were calculated using participant fasting blood glucose and insulin concentrations. Optimal insulin sensitivity is generally HOMA-IR less than 1; levels above 1.9 indicate early insulin resistance, while levels above 2.9 indicate significant insulin resistance.

MetS severity z score calculates an individual’s MetS severity using established and well-researched questions [34] and was determined using an internet-based MetS severity score calculator [35]. Scores were calculated using participant demographics (age, gender, ethnicity), anthropometric measurements (height, weight, waist circumference, systolic blood pressure) and laboratory measurements (HDL, triglycerides, and fasting glucose). If z score is above 0, this is associated with higher risk for future disease; scores above 1 are higher than 84% of US adults and scores above 2 are higher than 98% of US adults.

### 2.7. Physical Activity and Dietary Intake

Participants were asked to maintain their usual diets and physical activity and not to alter their lifestyle during the interventional period. Physical activity was monitored using the International Physical Activity Questionnaire (IPAQ) short form and reported as metabolic equivalent (MET) minutes/week. Low physical activity corresponds to <600 MET minutes/week; moderate physical activity corresponds to at least 600 MET minutes/week of moderate activities, and high physical activity corresponds to at least 1500 MET minutes/week of intense activities or at least 3000 MET minutes of moderate activities [36].

Recent dietary intakes were monitored using 24 h dietary recalls. These were used to calculate fruit intake (150 g servings), vegetable intake (75 g servings), and vitamin C intake (mg/d) using FoodWorks.online (Xyris, Brisbane, Australia). The recommended daily fruit and vegetable intakes in New Zealand are 2 servings of fruit and 5 to 6 servings of vegetables for females and males, respectively [37], and the recommended dietary intake for vitamin C is 45 mg/day [29].

### 2.8. Statistical Analyses

Participant characteristics were summarised using descriptive statistics and tabulated by treatment group. Continuous variables are presented as median and interquartile range (Q1, Q3) or mean and standard error of the mean (SEM) or mean and 95% confidence intervals (95%CI), as indicated, and categorical variables as number and percentage. Linear regression correlations were carried out using Pearson’s coefficient and non-parametric correlations using Spearman’s coefficient. Between-group comparisons were carried out using non-parametric Mann–Whitney U tests, with *p* < 0.05 indicating statistical significance. Sensitivity analyses investigated differences in outcomes according to treatment compliance/response as determined by vitamin C levels achieved. Participants taking insulin (*n* = 3) were excluded from analyses of insulin, HOMA-IR, HOMA%S and HOMA%B. Time course data were analysed using repeated measures mixed effects models (with Geisser–Greenhouse correction) and Tukey post hoc analyses to correct for multiple comparisons. Statistical analyses and graphical outputs were generated using GraphPad Prism 9 (GraphPad, San Diego, CA, USA).

## 3. Results

### 3.1. Participant Characteristics

**Figure 1:**
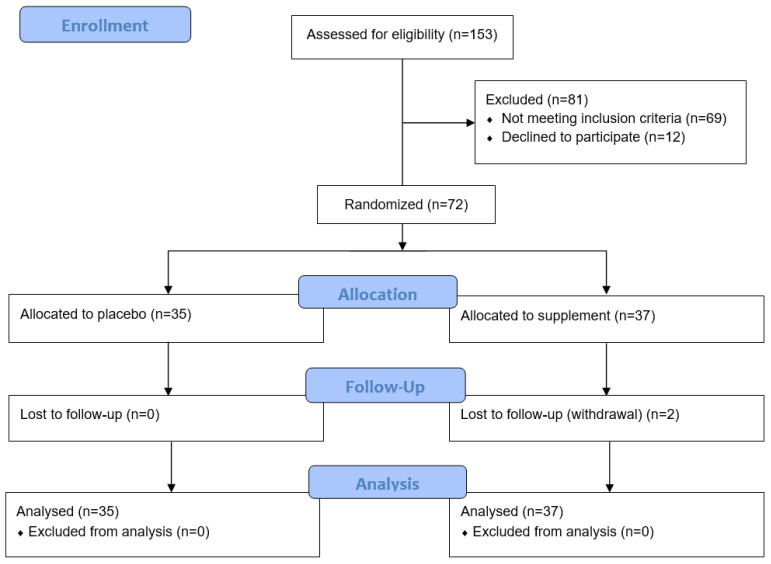
Consort flow diagram of participant recruitment and analysis.

Of the 153 participants screened for the study, 81 were excluded due to not meeting the inclusion criteria or declining to participate (Figure 1). Of the 72 randomised to the study, 37 were allocated to the micronutrient supplement group and 35 to the placebo group. Rolling recruitment allowed for the recruitment of an additional two participants to compensate for the two who withdrew during their study period.

The final cohort was predominantly female (86%), primarily European ethnicity (89%) and had a median (Q1, Q3) age of 52 (44, 63) years (range 27–76 years; Table 2). The cohort comprised predominantly non-smokers (97%). Over 60% of the cohort had hypertension and nearly 20% had diabetes; of these, over 70% were prescribed oral medication and/or insulin. The participants had been prescreened to have higher body weight and/or central obesity as indicated by their waist-to-hip ratio (Table 2). Furthermore, the participants had been prescreened to have elevated CRP concentrations (≥3 mg/L), reflected in the median baseline concentration of 5.6 (3.6, 8.8) mg/L (Table 3). The baseline values for the glycaemic markers (glucose, insulin and HbA1c) and lipid markers (triglycerides, LDL cholesterol, total cholesterol and HDL cholesterol) are also indicated in Table 3.

The recent dietary intake of the cohort was estimated using 24 h dietary recall and, at baseline, the cohort was found to have a low daily intake of fruit (0.4 [0, 1.3] 150 g servings) and vegetable (2.1 [0.7, 6.1] 75 g servings) resulting in a relatively low total fruit and vegetable intake of 3.2 (1.3, 6.9) servings per day (Table 2). The estimated median (Q1, Q3) vitamin C intake of the cohort was determined to be 43 (19, 85) mg/day (Table 2). Of these, 37% did not reach the NZ estimated average requirement (EAR; 30 mg/d), 54% did not reach the NZ recommended dietary intake (RDI; 45 mg/d) and 93% did not reach the NZ suggested dietary target for the reduction of chronic disease risk (SDT; 190 and 220 mg/d for females and males, respectively) [29]. The groups were well balanced in characteristics at baseline and there were no significant changes in fruit and vegetable intake over the duration of the study (*p* > 0.05).

**Figure 2:**
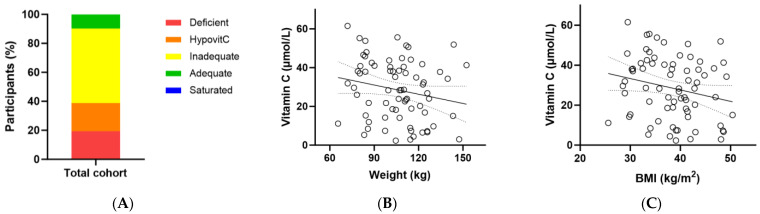
Baseline vitamin C status in the metabolic syndrome cohort (*n* = 72). (**A**) Baseline plasma vitamin C status was categorised as deficient (≤11 µmol/L), hypovitaminosis C (≤23 µmol/L), inadequate (<50 µmol/L), adequate (≥50 µmol/L), and saturated (≥70 µmol/L); (**B**) Correlation between baseline vitamin C status and body weight (r = −0.229, *p* = 0.05); (**C**) Correlation between baseline vitamin C status and BMI (r = −0.226, *p* = 0.06).

At baseline, the total cohort had a median (Q1, Q3) plasma vitamin C status of 29 (15, 41) µmol/L (Table 3). Of these, only 10% had adequate vitamin C concentrations (i.e., ≥50 µmol/L); 90% had inadequate concentrations (<50 µmol/L), 38% had hypovitaminosis C (≤23 µmol/L) and 19% had outright deficiency (≤11 µmol/L; Figure 2A). There was an inverse association between baseline vitamin C status and body weight (r = −0.229, *p* = 0.05) and a trend with BMI (r = −0.226, *p* = 0.06) (Figure 2B,C).

### 3.2. Effect of Intervention on Vitamin C Status

**Figure 3:**
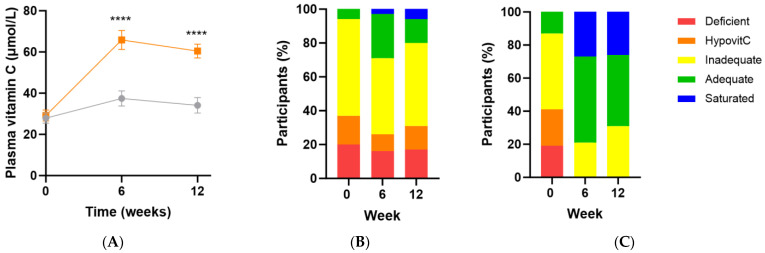
Changes in vitamin C status over time following intervention. (**A**) Changes over time in the micronutrient group (orange squares; *n* = 37) and placebo group (grey circles; *n* = 35); (**B**) Placebo group vitamin C categories over time; (**C**) Intervention group vitamin C categories over time. Plasma vitamin C status was categorised as deficient (≤11 µmol/L), hypovitaminosis C (≤23 µmol/L), inadequate (<50 µmol/L), adequate (≥50 µmol/L), and saturated (≥70 µmol/L). Symbols represent mean and SEM. **** *p* < 0.0001.

Following intervention there was a significant increase in vitamin C status in the micronutrient group relative to the placebo group (Δ26 [95%CI 16, 36] µmol/L; *p* < 0.0001), with a vitamin C concentration of 60 (47, 70) µmol/L achieved at week 12 (Figure 3A). There was an inverse correlation between baseline vitamin C status and the change in vitamin C concentration following intervention (r = −0.5 [95%CI −0.7, −0.2], *p* = 0.001), meaning the lower the baseline vitamin C status, the larger the increase following supplementation. Increased urinary excretion of the vitamin was also observed in the supplement group at weeks 6 and 12 (*p* ≤ 0.05), confirming saturation of the plasma. Of note though, approximately 20–30% of the participants did not reach adequate vitamin C status (i.e., 50 µmol/L) following supplementation (Figure 3C). These participants also had lower urinary excretion of the vitamin (*p* < 0.05). The attenuated response of these participants to supplementation (37 [32, 45] µmol/L vs. 63 [60, 79] µmol/L at 12 weeks, *p* < 0.0001) was predicted by lower vitamin C status at baseline (14 [7, 24] µmol/L vs. 36 [21, 47] µmol/L, *p* = 0.0005).

Baseline leukocyte vitamin C concentrations did not appear to be overly depleted, with a median (Q1, Q3) of 49 (38, 87) nmol/10^8^ cells, comparable to an earlier cohort with low vitamin C intake [38]. There was, however, no correlation between plasma and leukocyte vitamin C concentrations (r = 0.021, *p* = 0.9). Whilst leukocyte vitamin C concentrations increased in the intervention group relative to placebo following 12 weeks of supplementation (Δ12 [95%CI −8, 33] nmoles/10^8^ cells), this was not statistically significant (*p* = 0.35).

### 3.3. Effect of Intervention on Inflammatory and Oxidative Biomarkers

The baseline CRP concentration of the cohort was 5.6 (3.6, 8.8) mg/L and this did not change significantly following intervention with micronutrient formula relative to placebo (Δ−0.3 [95%CI −2.7, 2.1] mg/L, *p* = 0.8; Table 4). Subgroup analyses of those with low vitamin C status (<50 µmol/L or ≤23 µmol/L) or elevated CRP (>3 mg/L or >10 mg/L) did not significantly affect the results (*p* > 0.05). The baseline IL-6 concentration was 9.9 (6.7, 15) pg/mL and although the micronutrient group was trending towards a lower concentration over time (Δ−2.9 [95%CI −7.2, 1.4] pmol/L), this did not reach statistical significance (*p* = 0.18; Table 4). TNFα concentration at baseline was 0.48 (0, 3.3) pg/mL and this did not change significantly over time relative to placebo (*p* = 0.38; Table 4). Baseline F_2_isoprostane concentration was 1.4 (0.4, 2.3) ng/mg creatinine and the intervention group did not change significantly over time relative to placebo (*p* = 0.67; Table 4).

**Figure 4:**
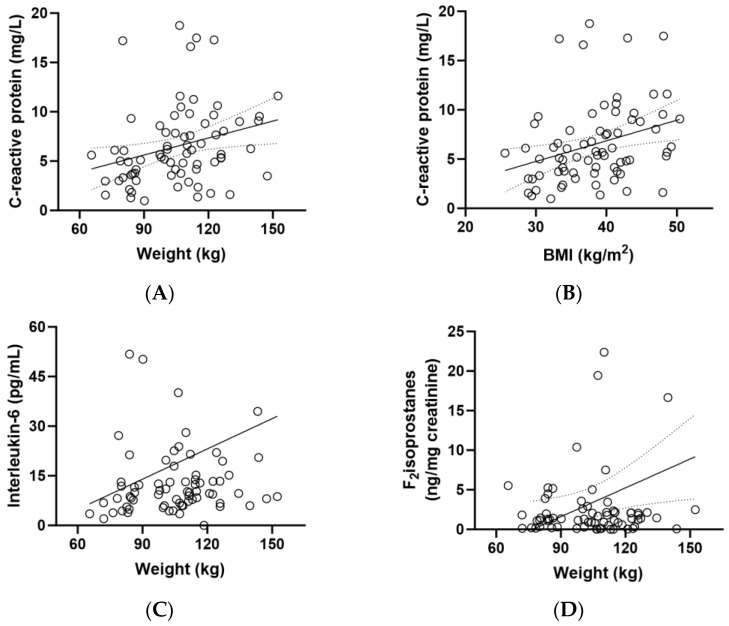
Correlations between biomarkers and weight (*n* = 72). (**A**) Correlation between baseline C-reactive protein and body weight (r = 0.330, *p* = 0.005); (**B**) correlation between baseline C-reactive protein and BMI (r = 0.361, *p* = 0.002); (**C**) correlation between baseline interleukin-6 and weight (0.229, *p* = 0.05); (**D**) correlation between baseline F_2_isoprostanes and weight (r = 0.268, *p* = 0.02).

CRP concentrations correlated closely with weight (r = 0.330, *p* = 0.005) and BMI (r = 0.361, *p* = 0.002) (Figure 4A,B). There was also a correlation between IL-6 concentrations and participant weight (*p* = 0.229, *p* = 0.05) and between F_2_isoprostane concentrations and weight (r = 0.268, *p* = 0.02; Figure 4C,D). Although there was a correlation between CRP and weight/BMI (Figure 4A,B) and an inverse correlation between vitamin C and weight/BMI (Figure 2B,C), there was no correlation between CRP and vitamin C (r = 0.120, *p* = 0.3).

### 3.4. Impact of Intervention on Weight Parameters

**Figure 5:**
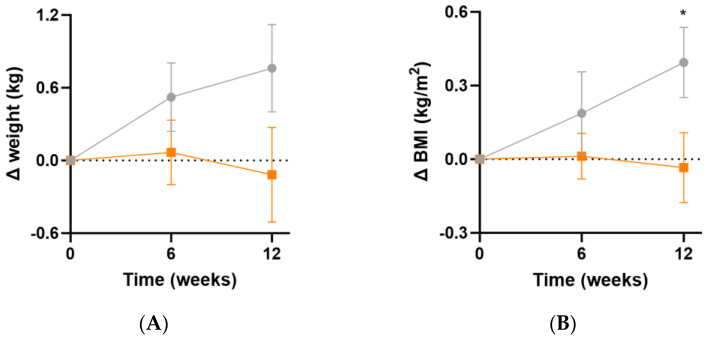
Changes in weight and BMI following intervention. (**A**) Changes in weight; (**B**) changes in BMI. Baseline weight and BMI can be found in Table 1. Orange squares, micronutrient group (*n* = 37); grey circles, placebo group (*n* = 35). Symbols represent mean and SEM. * *p* < 0.05.

At week 12, there were small increases in body weight (Δ0.9 [95%CI −0.2, 1.9] kg, *p* = 0.1) and BMI (Δ0.4 [95%CI 0.0, 0.8] kg/m^2^, *p* = 0.04) in the placebo group relative to the intervention group (Figure 5A,B). These changes were likely not sufficiently large to correspond with a measurable change in inflammatory or oxidative stress biomarkers. There was no significant change in waist-to-hip ratio between the two groups over the duration of the study (*p* > 0.05).

### 3.5. Effect of Intervention on Glycaemic and Lipid Parameters

**Figure 6:**
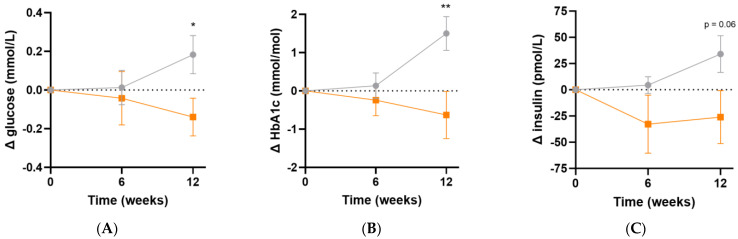
Changes in glycaemic indices following intervention. (**A**) Changes in fasting plasma glucose; (**B**) changes in glycated haemoglobin (HbA1c); (**C**) changes in fasting insulin concentrations; participants taking insulin (*n* = 3) were excluded from insulin values. Baseline values can be found in Table 2. Orange squares, micronutrient group (*n* = 34); grey circles, placebo group (*n* = 35). Symbols represent mean and SEM. * *p* < 0.05, ** *p* < 0.01.

Following micronutrient supplementation, there was a significant difference in fasting plasma glucose concentrations (Δ−0.3 [95%CI −0.6, −0.05] mmol/L, *p* = 0.02; Figure 6A) and HbA1c concentrations (Δ−2.1 [95%CI −3.6, −0.6] mmol/L, *p* = 0.007; Figure 6B) relative to the placebo group. Similarly, there was a trend towards lower fasting insulin concentrations relative to placebo following 12 weeks of intervention (Δ−60 [95%CI −122, 1.7] pmol/L, *p* = 0.06; Figure 6C). LDL cholesterol concentrations showed a trend towards a decline over the duration of the study relative to placebo (Δ−0.2 [95%CI −0.5, 0.1] mmol/L, *p* = 0.08). There were no significant differences in the other lipid markers (triglycerides, total cholesterol or HDL cholesterol) after 12 weeks of supplementation (*p* > 0.05, Table 5). There were no changes in systolic or diastolic blood pressure over the duration of the study (*p* > 0.05).

### 3.6. Effect of Intervention on HOMA Indices and MetS z Score

**Figure 7:**
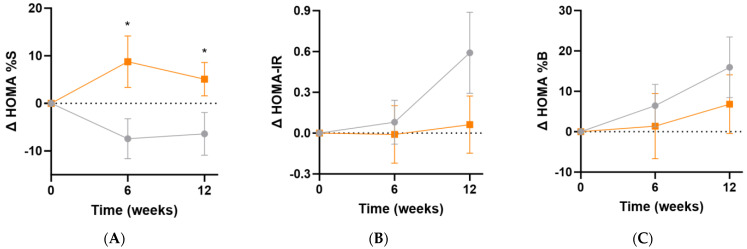
Changes in HOMA indices following intervention. (**A**) Changes in insulin sensitivity (baseline HOMA%S was 48 [32, 66]); (**B**) changes in insulin resistance (baseline HOMA-IR was 2.1 [1.5, 3.1]); (**C**) changes in ß-cell activity (baseline HOMA%B was 107 [87, 142]). Participants taking insulin (*n* = 3) were excluded from these analyses. Orange squares, micronutrient group (*n* = 34); grey circles, placebo group (*n* = 35). Symbols represent mean and SEM. * *p* < 0.05.

The Homeostatic Model Assessment of insulin sensitivity (HOMA%S) was shown to significantly improve in the micronutrient group following supplementation (Δ12 [95%CI 0.1, 23]%; *p* = 0.049), and this was apparent even at the 6-week point (Δ16 [95%CI 2.5, 30]%, *p* = 0.02; Figure 7A). Changes in insulin resistance (HOMA-IR) indicated a trend towards increased insulin resistance in the placebo group relative to the micronutrient group (Δ0.5 [95%CI −0.2, 1.2], *p* = 0.15; Figure 7B). There were no significant differences in ß-cell activity (HOMA%B) between the intervention and placebo groups at week 12 (Δ−9% [−30, 12]%, *p* = 0.39; Figure 7C).

**Figure 8:**
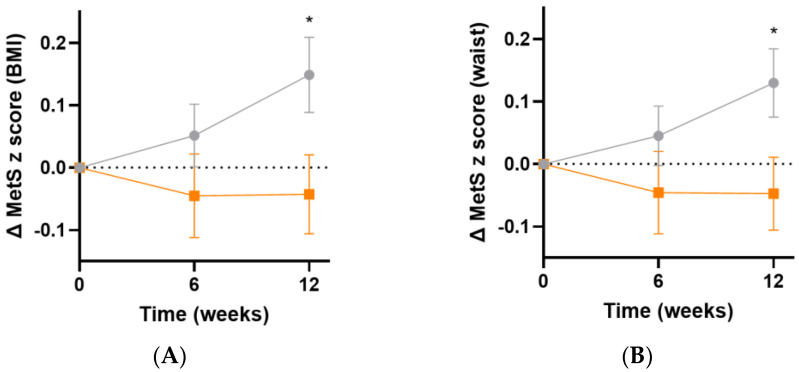
Changes in metabolic syndrome severity (MetS) z score following intervention. (**A**) Changes in MetS z score based on BMI (baseline value was 0.4 [0.05, 1.0]); (**B**) changes in MetS z score based on waist (baseline value was 0.4 [0.1, 0.9]). Orange squares, micronutrient group (*n* = 34); grey circles, placebo group (*n* = 35). Symbols represent mean and SEM. * *p* < 0.05.

The metabolic syndrome severity (MetS) z score is based on a number of parameters: sex, ethnicity, BMI or waist circumference, systolic blood pressure, and fasting glucose, triglyceride and HDL concentrations [35]. Following 12 weeks of intervention, there was an increase in MetS z score in the placebo group, but not in the micronutrient group, resulting in a difference of 0.2 (95%CI 0.02, 0.40), *p* = 0.03, based on BMI (Figure 8A) and 0.2 (95%CI 0.02, 0.3), *p* = 0.03, based on waist circumference (Figure 8B).

No adverse events attributable to micronutrient supplement consumption, other than more frequent urination (*n* = 1), were reported during follow-up.

## 4. Discussion

Our trial assessed the effect of a micronutrient supplement containing 1000 mg vitamin C on markers of inflammation in people with metabolic syndrome and elevated baseline inflammation. Our study showed low baseline vitamin C status and a high proportion of hypovitaminosis C and outright deficiency in people with metabolic syndrome relative to randomly selected community-dwelling participants from the CHALICE study [39]. Following 12 weeks of micronutrient supplementation, there was a significant increase in vitamin C status in the participants in the intervention arm relative to placebo. Despite this, there was no significant change in the inflammatory biomarker CRP, or the inflammatory cytokines IL-6 or TNFα, in agreement with other vitamin C/antioxidant intervention studies in obesity and diabetes [21,22]. The participants had been prescreened for CRP concentrations ≥3 mg/L, indicative of high risk for CVD. Ellulu et al. [20] also prescreened for elevated CRP and were able to show a decrease in CRP concentrations relative to control following vitamin C intervention. However, the baseline CRP concentrations in the current study were only half that of Ellulu et al. [20], indicating that their obese hypertensive and/or diabetic participants were more severe at baseline, and hence more likely to respond to intervention [40].

The lipid oxidation marker, F_2_isoprostane, was elevated at baseline in the metabolic syndrome cohort relative to healthy non-smoking controls, although not as high as that of smokers [41]. In contrast to Murer et al. [22], who reported a decrease in urinary F_2_isoprostanes following four-month antioxidant supplementation in obese children and adolescents, the urinary F_2_isoprostane concentrations did not change following 12 weeks of micronutrient supplementation in the metabolic syndrome cohort. Although protein carbonyls were assessed as another potential marker of oxidative stress, these were not elevated at baseline in the participants with metabolic syndrome relative to healthy controls so were not pursued.

Small improvements were observed in glycaemic indices (fasting glucose and HbA1c concentrations) and indices of metabolic health (insulin sensitivity and metabolic severity score) in the group who received the micronutrient supplement relative to the placebo group. Meta-analyses have indicated that supplementation with vitamin C can improve markers of metabolic health (glycaemic control and lipid profiles), particularly in people with low vitamin C status or dysregulated metabolic markers at baseline [42,43]. We furthermore observed a stabilisation of weight and BMI relative to the placebo group which continued to increase over the duration of the study. These findings are supported by previous RCTs investigating supplementation of people with metabolic syndrome with 500 mg/d vitamin C which indicated decreased BMI and improved lipid profiles compared with the placebo group [44,45]. Metabolic outcomes may also be improved with a combination of physical activity and vitamin C supplementation [44,45], with antioxidant supplementation potentially attenuating exercise-induced oxidative stress and inflammation in overweight and diabetic individuals [46,47].

The mechanisms of action for these metabolic health improvements are currently uncertain as we were not able to demonstrate an overall effect of the micronutrient formula on systemic markers of inflammation and oxidative stress. Nevertheless, the vitamins and minerals present in the micronutrient formula have vital coenzyme or cofactor roles in numerous important physiological processes including metabolic health [48]. Vitamin C, in particular, has enzyme cofactor functions that facilitate mitochondrial lipid utilisation, as well as the synthesis of amidated peptide hormones vital to the digestive system and metabolic health [49].

Of note was the strong association between the inflammatory marker CRP and body weight and BMI in the study participants, which has previously been reported in people with diabetes relative to those without [50]. Similar associations were observed for the other inflammatory and oxidative stress biomarkers (IL-6 and F_2_isoprostanes). As such, the relatively small improvement in weight and BMI in the micronutrient group relative to the placebo group may have been insufficient to translate to a measurable improvement in systemic markers of inflammation and oxidative stress. Although vitamin C intervention has previously been reported to decrease CRP concentrations, particularly in those with elevated CRP at baseline [40], it is not certain if body weight was considered a confounding factor in these studies, in that any changes in body weight would likely impact on the CRP concentrations measured. Of note, there were no correlations between vitamin C and CRP concentrations at baseline in our metabolic syndrome cohort. Thus, future studies investigating more closely the associations between vitamin C, CRP and weight/BMI appear warranted as changes in CRP may simply be reflecting changes in body weight/obesity, rather than a direct effect of vitamin C on CRP status per se.

Approximately 20–30% of the cohort did not achieve adequate vitamin C status following supplementation with 1000 mg/day of the vitamin. This subgroup of participants had a steady state vitamin C status at 12 weeks that was <40 µmol/L vs. >60 µmol/L. Low baseline vitamin C status was found to predict decreased response to supplementation, which has been noted previously [15,51]. Overall, having low baseline vitamin C status and an attenuated response to supplementation likely reflects long-term depletion of body stores which may require either higher doses or longer duration of intervention to fully replete the depleted tissues. There was a strong inverse correlation between baseline vitamin C status and weight/BMI, which has been reported previously [17], and higher body weight has also been shown to result in a higher requirement for the vitamin [15,16]. Since people with obesity and diabetes tend to have both a depleted nutrient status and a significantly increased risk, morbidity and mortality from infections, including COVID-19 [52,53], ensuring adequate concentrations of immune-supportive nutrients is vital in these vulnerable subgroups within the population [54]. The small improvements in weight and metabolic health indices observed in this study may indirectly contribute to improved immunity in people with metabolic syndrome, particularly if continued supplementation is able to provide even larger improvements.

The strengths of the study included the gold-standard double-blind, randomized placebo-controlled study design, the use of gold-standard analytical techniques, and a well-characterised cohort that was prescreened for high inflammation at baseline, thus enhancing the likelihood of detecting an effect of the intervention. A limitation of the study was the low proportion of males recruited, which may limit the generalizability of the findings. Another limitation was the lack of prescreening for low vitamin C status, although many participants, by the very nature of their condition, had inadequate vitamin C status at baseline. Future studies could prescreen for hypovitaminosis C at baseline and undertake dose-finding to determine the optimal intervention dosing for people with metabolic syndrome and low baseline vitamin C status. A further limitation was the use of a multinutrient supplement, which makes it difficult to ascertain which of the micronutrients are having the positive effects. For example, other micronutrients in the formula also have beneficial effects in obesity, e.g., vitamins A, B6 and B12, and selenium [23,24,25,26]. Furthermore, micronutrients can have synergistic activities, such as vitamins C and E, whereby vitamin C facilitates vitamin E’s lipid antioxidant effects [55,56]. Although no evidence for a lipid antioxidant effect was observed in this study (lack of overall change in F_2_isoprostane concentrations), the concentration of vitamin E in the micronutrient supplement was significantly lower than that of vitamin C (i.e., only 4.5%). As such, higher concentrations of vitamin E may be required for synergistic antioxidant effects. Finally, the cohort was relatively diverse with various types and severities of metabolic dysregulation. Thus, it is possible that higher doses of micronutrients may be required for those with more severe dysregulation.

## 5. Conclusions

Inflammatory CRP concentrations were closely tied to body weight and BMI, and micronutrient supplementation alone was unable to mitigate this weight-associated inflammation. As such, combination therapies such as exercise (or supplements that aid in weight loss) plus micronutrients (to replenish depleted nutrient status) may provide better outcomes by attenuating the weight gain and associated inflammation that is linked to the progression of metabolic syndrome to type 2 diabetes and CVD.

## Figures and Tables

**Table 1:** Micronutrient content of effervescent tablets.

Micronutrients	Daily Dose	NZ RDI/AI *^a^*	NZ UL *^a^*
Vitamins:			
Vitamin C (mg)	1000	45	ND
Vitamin E (mg)	45	7	300
Vitamin B6 (mg)	6.5	1.3	50
Vitamin A (µg)	700	700	3000
Folate, B9 (µg)	400	400	1000
Vitamin D (µg)	10	5	80
Vitamin B12 (µg)	9.6	2.4	ND
Trace minerals:			
Zinc (mg)	10	8	40
Iron (mg)	5	8–18	45
Copper (mg)	0.9	1.2	25
Selenium (µg)	110	60	400

*^a^* Values from [29]. Abbreviations: RDI, recommended dietary intake (for females); AI, adequate intake (for females); UL, upper level of intake; ND not able to be determined.

**Table 2:** Participant demographic and anthropometric characteristics.

Parameter	Total Cohort (*n* = 72)	Placebo Group (*n* = 35)	Micronutrient Group (*n* = 37)
Age (years)	52 (44, 63)	51 (42, 63)	52 (48, 63)
Sex (female)	62 (86)	32 (91)	30 (81)
Ethnicity:			
NZ European	57 (79)	28 (80)	29 (78)
Māori/Pacific	9 (13)	4 (12)	5 (13)
Other European	7 (10)	1 (3)	6 (16)
Asian	3 (4)	2 (6)	2 (5)
African	1 (1)	1 (3)	0 (0)
Smoking status:			
Never smoked	66 (92)	33 (94)	33 (89)
Former smoker	4 (5)	1 (3)	3 (8)
Current smoker	2 (3)	1 (3)	1 (3)
Taking medications:			
Diabetes *^a^*	10 (14)	3 (9)	7 (19)
Hypertension *^b^*	36 (50)	17 (49)	19 (51)
Hyperlipidaemia *^c^*	17 (24)	9 (26)	8 (22)
Others	53 (74)	25 (71)	28 (76)
Systolic BP (mmHg)	133 (123, 143)	132 (123, 138)	133 (124, 147)
Diastolic BP (mmHg)	84 (78, 91)	85 (80, 91)	83 (78, 89)
Weight (kg)	107 (86, 118)	107 (84, 117)	110 (87, 120)
BMI (kg/m^2^)	39 (34, 42)	39 (34, 44)	38 (34, 42)
Waist-hip ratio	0.90 (0.7, 0.93)	0.89 (0.87, 0.92)	0.90 (0.87, 0.94)
Physical activity (MET minutes/week)	1664 (652, 3965)	2079 (897, 5424)	1520 (650, 3132)
Fruit & vegetable intake (servings/d) *^d^*	3.2 (1.3, 6.9)	2.6 (1.1, 6.2)	3.7 (1.3, 7.6)
Vitamin C intake (mg/d)	43 (19, 85)	30 (12, 86)	48 (21, 85)

Data represent median (Q1, Q3) or n (%). *^a^* Diabetes medications: Insulin, Metformin, Dulaglutide, Empagliflozin, Vildagliptin. *^b^* Hypertension medications: Angiotensin converting enzyme (ACE) inhibitors (Cilazapril, Enalapril, Lisinopril, Perindopril, Quinapril), Angiotensin receptor blockers (ARB; Candesartan, Losartan), Beta blockers (Bisoprolol, Metoprolol, Nadolol, Propranolol), Calcium channel blockers (Amlodipine, Diltiazem, Felodipine), Diuretics (Chlorthalidone, Indapamide). *^c^* Hyperlipidaemia medications: Cholesterol-absorption inhibitors (Ezetimibe), HMG-CoA reductase inhibitors (Atorvastatin, Pravastatin). *^d^* Serving is equivalent to 150 g fruit or 75 g vegetable [37]. BMI, body mass index; BP, blood pressure; MET, metabolic equivalent of task.

**Table 3:** Participant baseline laboratory parameters.

Parameter	Total Cohort (*n* = 72)	Placebo Group (*n* = 35)	Micronutrient Group (*n* = 37)
C-reactive protein (mg/L)	5.6 (3.6, 8.8)	5.7 (3.6, 9.1)	5.3 (3.7, 8.3)
Vitamin C (µmol/L)	29 (15, 41)	28 (15, 38)	29 (16, 43)
Glucose (mmol/L)	5.8 (5.4, 6.9)	5.8 (5.5, 6.7)	5.9 (5.3, 6.6)
Insulin (pmol/L) *^a^*	102 (78, 154)	101 (79, 151)	112 (80, 175)
HbA1c (mmol/mol)	40 (37, 42)	40 (37, 42)	40 (37, 44)
Triglycerides (mmol/L)	1.5 (1.1, 2.0)	1.3 (1.0, 1.9)	1.5 (1.2, 2.5)
Total cholesterol (mmol/L)	5.3 (4.6, 5.9)	5.4 (4.6, 5.7)	5.3 (4.7, 6.1)
LDL cholesterol (mmol/L)	3.1 (2.7, 3.8)	3.1 (2.6, 3.8)	3.0 (2.8, 3.8)
HDL cholesterol (mmol/L)	1.3 (1.2, 1.5)	1.4 (1.2, 1.6)	1.2 (1.1, 1.5)

Data represent median (Q1, Q3). *^a^* Participants taking insulin (*n* = 3) were excluded. HbA1c, glycated haemoglobin; LDL, low-density lipoprotein; HDL, high-density lipoprotein.

**Table 4:** Effect of intervention on inflammatory and oxidative biomarkers.

	Placebo Group (*n* = 35)			Micronutrient Group (*n* = 37)			Between Group Delta *^b^*
Biomarker	Baseline	Week 12	Delta	Baseline	Week 12	Delta	
CRP (mg/L)	5.7 (3.6, 9.1)	5.8 (3.0, 7.7)	−0.1 (−2.5, 2.4)	5.3 (3.7, 8.3)	6.1 (4.2, 8.7)	0.5 (−1.0, 1.9)	−0.3 (−2.7, 2.1)
IL-6 (pg/mL) *^a^*	8.7 (5.4, 13)	9.6 (5.4, 15)	0.6 (−1.9, 3.0)	11 (7.9, 20)	10 (7.1, 17)	−2.5 (−7.1, 2.0)	−2.9 (−7.2, 1.4)
TNFα (pg/mL)	0.6 (0.0, 3.2)	1.4 (0.9, 2.6)	0.2 (−0.4, 0.7)	1.0 (0.0, 4.1)	2.2 (0.3, 3.5)	0.3 (−0.3, 0.9)	0.1 (−0.6, 0.8)
F_2_isoprostanes (pg/mg creatinine)	1.4 (0.9, 2.6)	1.5 (1.0, 3.1)	−0.3 (−2.7, 2.0)	1.4 (0.3, 2.3)	1.3 (1.0, 2.2)	−1.9 (−6.6, 2.7)	−1.8 (−5.9, 2.4)

Data represent median (Q1, Q3) or mean (95%CI) for delta. *^a^* One significantly outlying data point was excluded in the intervention group at baseline and one in the placebo group at 12 weeks. *^b^* Estimated between-group difference (delta) was corrected for baseline. There were no significant differences between the groups for any of the biomarkers (*p* > 0.05).

**Table 5:** Effect of intervention on lipid parameters.

	Placebo Group (*n* = 35)			Micronutrient Group (*n* = 37)			Between Group Delta *^a^*
Biomarker	Baseline	Week 12	Delta	Baseline	Week 12	Delta	
Triglycerides (mmol/L)	1.3 (1.0, 1.9)	1.4 (1.0, 2.2)	0.1 (−0.1, 0.4)	1.5 (1.2, 2.5)	1.8 (1.2, 2.4)	0.1 (−0.3, 0.4)	−0.0 (−0.3, 0.3)
Total cholesterol (mmol/L)	5.4 (4.6, 5.7)	5.2 (4.2, 6.3)	0.1 (−0.2, 0.3)	5.3 (4.7, 6.1)	5.2 (4.3, 6.2)	−0.1 (−0.4, 0.2)	−0.1 (−0.5, 0.2)
LDL cholesterol (mmol/L)	3.1 (2.6, 3.8)	3.3 (2.7, 3.9)	0.0 (−0.2, 0.2)	3.0 (2.8, 3.8)	3.0 (2.5, 3.5)	−0.2 (−0.5, 0.1)	−0.2 (−0.5, 0.1)
HDL cholesterol (mmol/L)	1.4 (1.2, 1.6)	1.4 (1.2, 1.6)	0.0 (−0.1, 0.1)	1.2 (1.1, 1.5)	1.3 (1.1, 1.4)	0.0 (−0.1, 0.1)	0.0 (−0.1, 0.1)

Data represent median (Q1, Q3) or mean (95%CI) for delta. *^a^* Estimated between-group difference (delta) was corrected for baseline. There were no significant differences between the groups for any of the biomarkers (*p* > 0.05). LDL, low-density lipoprotein; HDL, high-density lipoprotein.

## Data Availability

Data described in the manuscript will be made available upon reasonable request pending application and approval.
